# Supplementary material for: Sensitivity to TDP-43 loss and degradation resistance determine cryptic exon biomarker potential
Source: bioRxiv. 2025 Nov 23:2025.11.23.689722. Preprint. [Version 1] doi: 10.1101/2025.11.23.689722 (PMC12667841; doi:10.1101/2025.11.23.689722)
Supplement: 1 [file NIHPP2025.11.23.689722V1-supplement-1.pdf]

bioRxiv preprint doi: https://doi.org/10.1101/2025.11.23.689722; this version posted November 23, 2025. The copyright holder has placed this preprint (which was not certified by peer review) in the Public Domain. It is no longer restricted by copyright. Anyone can legally share, reuse, remix, or adapt this material for any purpose without crediting the original authors.

Tables

Supplementary Table 1. Gene expression changes in all TDP-43 knockdown datasets.

Supplementary Table 2. Summary of all detected cryptic including postmortem detection, TDP-43 sensitivity, and NMD sensitivity.

Supplementary Table 3. Summary of samples from NYGC and RiMOD cohorts.

bioRxiv preprint doi: https://doi.org/10.1101/2025.11.23.689722; this version posted November 23, 2025. The copyright holder has placed this preprint (which was not certified by peer review) in the Public Domain. It is no longer restricted by copyright. Anyone can legally share, reuse, remix, or adapt this material for any purpose without crediting the original authors.

Proteinopathy	Tissue Source	Samples (n)	Patients (n)	Dataset
Control	Cortex_Frontal	84	80	NYGC FTD Frontal/Temporal
Control	Cortex_Temporal	27	27	NYGC FTD Frontal/Temporal
FTLD-FUS	Cortex_Frontal	5	5	NYGC FTD Frontal/Temporal
FTLD-FUS	Cortex_Temporal	3	3	NYGC FTD Frontal/Temporal
FTLD-TAU	Cortex_Frontal	6	6	NYGC FTD Frontal/Temporal
FTLD-TAU	Cortex_Temporal	5	5	NYGC FTD Frontal/Temporal
FTLD-TDP	Cortex_Frontal	37	37	NYGC FTD Frontal/Temporal
FTLD-TDP	Cortex_Temporal	33	33	NYGC FTD Frontal/Temporal
ALS-TDP	Cortex_Motor	445	313	NYGC ALS motor cortex
ALS-non-TDP	Cortex_Motor	18	12	NYGC ALS motor cortex
Control	Cortex_Motor	71	48	NYGC ALS motor cortex
ALS-TDP	Cord_Spinal	704	349	NYGC ALS spinal cord
ALS-non-TDP	Cord_Spinal	27	13	NYGC ALS spinal cord
Control	Cord_Spinal	126	74	NYGC ALS spinal cord
Control	Cortex_Frontal	16	16	RiMOD FTD
FTLD-TAU	Cortex_Frontal	11	11	RiMOD FTD
FTLD-TDP	Cortex_Frontal	20	20	RiMOD FTD

378
